# Characterization of a *bla*_NDM-1_-Bearing IncHI5-Like Plasmid From *Klebsiella pneumoniae* of Infant Origin

**DOI:** 10.3389/fcimb.2021.738053

**Published:** 2021-10-01

**Authors:** Ziyi Liu, Ruifei Chen, Poshi Xu, Zhiqiang Wang, Ruichao Li

**Affiliations:** ^1^ Jiangsu Co-Innovation Center for Prevention and Control of Important Animal Infectious Diseases and Zoonoses, College of Veterinary Medicine, Yangzhou University, Yangzhou, China; ^2^ Institute of Comparative Medicine, Yangzhou University, Yangzhou, China; ^3^ Department of Clinical Laboratory, Henan Provincial People’s Hospital, Zhengzhou, China; ^4^ Department of Clinical Laboratory of Central China Fuwai Hospital, Central China Fuwai Hospital of Zhengzhou University, Zhengzhou, China

**Keywords:** IncHI5-like plasmid, *Klebsiella pneumoniae*, *bla*_NDM-1_, nanopore sequencing, evolution

## Abstract

The spread of plasmid-mediated carbapenem-resistant clinical isolates is a serious threat to global health. In this study, an emerging NDM-encoding IncHI5-like plasmid from *Klebsiella pneumoniae* of infant patient origin was characterized, and the plasmid was compared to the available IncHI5-like plasmids to better understand the genetic composition and evolution of this emerging plasmid. Clinical isolate C39 was identified as *K. pneumoniae* and belonged to the ST37 and KL15 serotype. Whole genome sequencing (WGS) and analysis revealed that it harbored two plasmids, one of which was a large IncHI5-like plasmid pC39-334kb encoding a wide variety of antimicrobial resistance genes clustered in a single multidrug resistance (MDR) region. The *bla*_NDM-1_ gene was located on a ΔIS*Aba125*-*bla*_NDM-1_-*ble*_MBL_-*trpF*-*dsbC* structure. Comparative genomic analysis showed that it shared a similar backbone with four IncHI5-like plasmids and the IncHI5 plasmid pNDM-1-EC12, and these six plasmids differed from typical IncHI5 plasmids. The replication genes of IncHI5-like plasmids shared 97.06% (*repHI5B*) and 97.99% (*repFIB-like*) nucleotide identity with those of IncHI5 plasmids. Given that pNDM-1-EC12 and all IncHI5-like plasmids are closely related genetically, the occurrence of IncHI5-like plasmid is likely associated with the mutation of the replication genes of pNDM-1-EC12-like IncHI5 plasmids. All available IncHI5-like plasmids harbored 262 core genes encoding replication and maintenance functions and carried distinct MDR regions. Furthermore, 80% of them (4/5) were found in *K. pneumoniae* from Chinese nosocomial settings. To conclude, this study expands our knowledge of the evolution history of IncHI5-like plasmids, and more attention should be paid to track the evolution pathway of them among clinical, animal, and environmental settings.

## Introduction

IncHI plasmids are important vectors in the dissemination of heavy metal resistance genes and antimicrobial resistance genes (Cain and Hall, 2012). These plasmids are usually larger than 200 kb and have a wide host range (Rozwandowicz et al., 2018). Members of the IncHI group contained five types of plasmids (Liang et al., 2018), of which considerable genetic conservation was detected within subgroups, but the conserved sequences of each subgroup were dramatically distinguished from each other (Liang et al., 2017). IncHI5 plasmids are associated with various carbapenemase genes (Zhu et al., 2020), implying that investigations are needed to continuously monitor the prevalence of IncHI5 plasmids in different sources, especially among clinical settings. More seriously, a recent study reported two IncHI5-like plasmids co-harboring carbapenem resistance genes and tigecycline resistance module *tmexCD1-toprJ1* (Qin et al., 2021), indicating that the IncHI5 plasmid has evolved and may develop as key vectors to carry important and novel antimicrobial resistance determinants. However, studies on the structures and evolution of IncHI5-like plasmids in pathogens remain limited. Currently, only five fully sequenced IncHI5-like plasmids are available (**Table 2**) (last accessed May 26, 2021). In this study, we aim to characterize a *bla*_NDM-1_ carrying IncHI5-like plasmid from *K. pneumoniae* and further illustrate the underlying evolution process by analysis of available sequenced IncHI5-like plasmids.

## Materials and Methods

### Sample Information and Characterization

*Klebsiella pneumoniae* C39 was isolated from a sputum sample of an infant patient with congenital heart disease and multiple complications in Henan, China in August 2019, using 5% sheep blood agar (see **Supplementary Table S1** for more information). The identification of bacterial species was conducted by 16S rRNA gene sequencing. The presence of carbapenemase genes was screened for multiplex PCR as previously mentioned (Poirel et al., 2011). The minimal inhibitory concentrations (MICs) of meropenem, ampicillin, ceftiofur, tetracycline, kanamycin, enrofloxacin, tigecycline, and colistin were determined by the broth microdilution method recommended by the Clinical and Laboratory Standards Institute (CLSI, M100-S28, 2018), while the MICs of the remaining 14 antimicrobials levofloxacin, chloramphenicol, aztreonam, ampicillin-sulbactam, piperacillin, piperacillin-tazobactam, gentamicin, amikacin, cefazolin, ceftazidime, cefotaxime, cefepime, imipenem, and trimethoprim-sulfamethoxazole were obtained by BD Phoenix100 (Becton, Dickinson and Company, Franklin Lakes, NJ, USA). The MIC breakpoints for Enterobacterales (susceptible, ≤2 mg/L; resistant, ≥8 mg/L) defined by the Food and Drug Administration were used for tigecycline. To investigate the transferability of *bla*_NDM-1_-bearing plasmid, filter mating and electrotransformation assays were performed using hygromycin-resistant *K. pneumoniae* YZ6 (Li et al., 2020) as the recipient strain, of which the conjugation assay was conducted at different temperatures (25°C, 30°C, and 37°C). Transconjugants or transformants were selected on LB agar plates supplemented with hygromycin (200 mg/L) and meropenem (2 mg/L).

### Genome Extractions and High-Throughput Sequencing

Genomic DNA was extracted using the TIANamp bacterial DNA kit (TianGen, Beijing, China), quantified by Qubit and gel electrophoresis and subjected to sequencing using the Oxford Nanopore Technologies MinION long-read platform in combination with the Illumina Hiseq system as described previously (Wick et al., 2017).

### Bioinformatics Analysis and Phylogenetic Tree Construction

Genome sequence of C39 was *de novo* assembled by Illumina short-read and Nanopore long-read MinION sequencing data. Gene annotations were carried out by RAST (http://rast.nmpdr.org/) automatically and modified manually. The sequence types (STs), plasmid replicon, insertion sequences, and antimicrobial resistance determinants of C39 were identified using Online tools (https://cge.cbs.dtu.dk/services/) and Kleborate (Wick et al., 2018). The BLAST Ring Image Generator tool and EasyFig served to generate the genetic comparison figures (Alikhan et al., 2011; Sullivan et al., 2011). Variant calls for single-nucleotide polymorphism (SNP) analysis were performed by Snippy (Seemann, 2014) against the sequence of pYNKP001-dfrA as it was the first identified IncHI5 plasmid, and SNPs on recombination sites were removed by Gubbins (Croucher et al., 2015). The filtered SNPs were then used as input for constructing a phylogenetic tree by FastTree (Price et al., 2009). The phylogenetic tree was visualized using interactive tree of life (ITOL) (Letunic and Bork, 2021). The core and pan genomes of all IncHI5-like plasmids were analyzed by Prokka and Roary (Seemann, 2014; Page et al., 2015). Combining the formed tree file and the gene presence and absence file, a plasmid phylogenetic tree with a matrix describing the presence and absence of core and accessory genes was constructed.

## Results and Discussion

### Strain Characteristics, Resistance Phenotypes, and Plasmid Transfer

*K. pneumoniae* C39 showed MDR phenotype, and β-lactam resistance to meropenem, ampicillin, ceftiofur, aztreonam, ampicillin-sulbactam, piperacillin, piperacillin-tazobactam, cefazolin, ceftazidime, cefotaxime, cefepime, and imipenem (**Table 1**). In addition, tetracycline, enrofloxacin, kanamycin, levofloxacin, chloramphenicol, amikacin, colistin, and tigecycline showed effective antibacterial activity against C39. Despite multiple attempts, carbapenem resistance phenotype could not transfer by conjugation or electrotransformation, indicating that the *bla*_NDM-1_ gene may be located on a nonconjugative plasmid.

**Table 1:** Antimicrobial susceptibility profiles of the *K. pneumoniae* strain C39 against different antimicrobials.

Strains	Antimicrobials (mg/L)^a^																					
	MEM	TIG	AMP	CFF	TET	KAN	ENR	CL	LVX	CHL	ATM	SAM	PIP	TZP	GEN	AMK	CZO	CAZ	CTX	FEP	IPM	SXT
C39	32	0.5	>256	>64	2	2	≤0.5	0.25	≤1	≤4	>16	>16	>64	>64	>8	≤8	>16	>16	>32	>16	>8	>2/38

a MEM, meropenem; TIG, tigecycline; AMP, ampicillin; CFF, ceftiofur; TET, tetracycline; KAN, kanamycin; ENR, enrofloxacin; CL, colistin; LVX, levofloxacin; CHL, chloramphenicol; ATM, aztreonam; SAM, ampicillin-sulbactam; PIP, piperacillin; TZP, piperacillin-tazobactam; Gen, gentamicin; AMK, amikacin; CZO, cefazolin; CAZ, ceftazidime; CTX, cefotaxime; FEP, cefepime; IPM, imipenem; SXT, cotrimoxazole trimethoprim-sulfamethoxazole.

### Characterization of *bla*_NDM-1_-Bearing Plasmid pC39-334kb

Strain C39 belonged to ST37 and KL15 serotype, and possessed multiple antimicrobial resistance genes encoding resistance to β-lactams (*bla*_SFO-1_-like, *bla*_VEB-3_, *bla*_TEM-1B_, *bla*_NDM-1_, and *bla*_SHV-11_), aminoglycosides [*aac(3’)-IId*], macrolides [*mph*(A)], quinolones (*qnrA7*, *oqxA*, and *oqxB*), sulphonamides (*sul1*), trimethoprim (*dfrA27*), rifampin (*arr-3*), bleomycin (*bla*_MBL_), and fosfomycin (*fosA*). ST37 *K. pneumoniae* is a clinically important pathogen frequently detected to carry *bla*_NDM_ (Zhang et al., 2019). Previously, two cases of pediatric patients that died due to *K. pneumoniae* ST37 infection were reported (Zhu et al., 2016), suggesting that the infant patients were suffering from the infection of high-risk pathogens. C39 contained one chromosome (5,290,020 bp, CP061700) and two plasmids pC39-334kb (334,893 bp, CP061701) and pC39-125kb (125,663 bp, CP061702) (**Supplementary Table S1**). pC39-125kb belonged to IncFIB replicon type, and no antimicrobial resistance (AMR) genes were found in this plasmid (**Supplementary Figure S1**).

**Figure 1:**
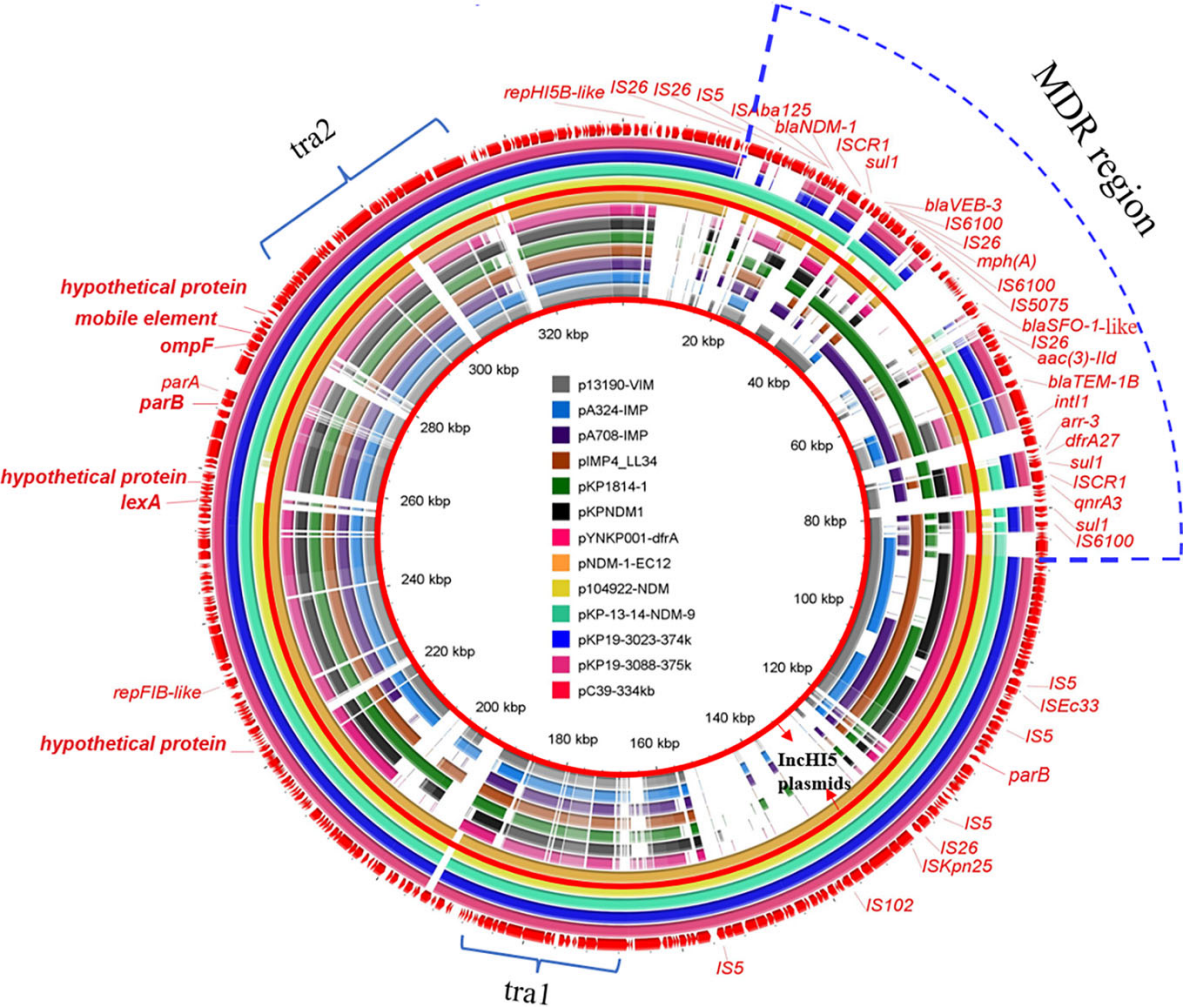
Circular comparison of pC39-334kb and other similar plasmids in the NCBI database. The outermost circle with red arrows represents the reference plasmid pC39-334kb. IncHI5 plasmids are shown between red concentric circles, and the MDR region of pC39-334kb is marked by a blue dotted line.

pC39-334kb was 334,893 bp in length with 379 predicted ORFs. Online BLAST against the NCBI nr database revealed that it shared an overall similar backbone with plasmid pNDM-1-EC12 (MN598004), pKP-13-14-NDM-9 (MN175386), p104922-NDM (MT062912), pKP19-3023-374kb (CP063748) and pKP19-3088-375kb (CP063149), including genes essential for replication (*repHI5B* and *repFIB-like*), partition (*parAB*) and conjugal transfer (tra1 and tra2) (**Figure 1**). As previously reported, pNDM-1-EC12 was identified as an IncHI5 plasmid (Zhu et al., 2020), whereas pKP19-3023-374kb and pKP19-3088-375kb belonged to IncHI5-like plasmids (Qin et al., 2021). The replication genes of IncHI5-like plasmids shared 97.06% (*repHI5B*) and 97.99% (*repFIB-like*) nucleotide identity with those of IncHI5 plasmids, while the repHI5B protein and repFIB-like protein of IncHI5-like plasmids shared 97.97% and 97.27% amino acid identity with those of IncHI5 plasmids. Therefore, pC39-334kb was classified as the IncHI5-like plasmid based on the comparative analysis of replication genes. Moreover, pC39-334kb displayed > 53% query coverage and > 98% identity to other seven representative IncHI5 plasmids pYNKP001-dfrA (KY270853), pKPNDM1 (JX515588), pKP1814-1 (KX839204), pIMP4_LL34 (CP025964), pA708-IMP (MF344567), pA324-IMP (MF344566) and p13190-VIM (MF344563), indicating that IncHI5-like plasmid shared some conserved sequences with IncHI5 plasmids. However, *lexA* (transcriptional regulator), *ompF* (outer membrane porin), mobile elements and several hypothetical genes located on the backbone of IncHI5-like plasmid were absent in seven IncHI5 plasmids (**Figure 1**).

The backbones of IncHI5 plasmids were diverse, where the integration of a wealth of accessory modules could be observed (Liang et al., 2018), so it was not surprised that the backbone of plasmid pNDM-1-EC12 exhibited differences with other IncHI5 plasmids. Considering that the backbone of pNDM-1-EC12 is almost identical to that of IncHI5-like plasmids, the IncHI5-like plasmid may originate from pNDM-1-EC12-like IncHI5 plasmids.

### Phylogenetic Analysis of pC39-334kb and IncHI5 Family Plasmids

**Figure 2:**
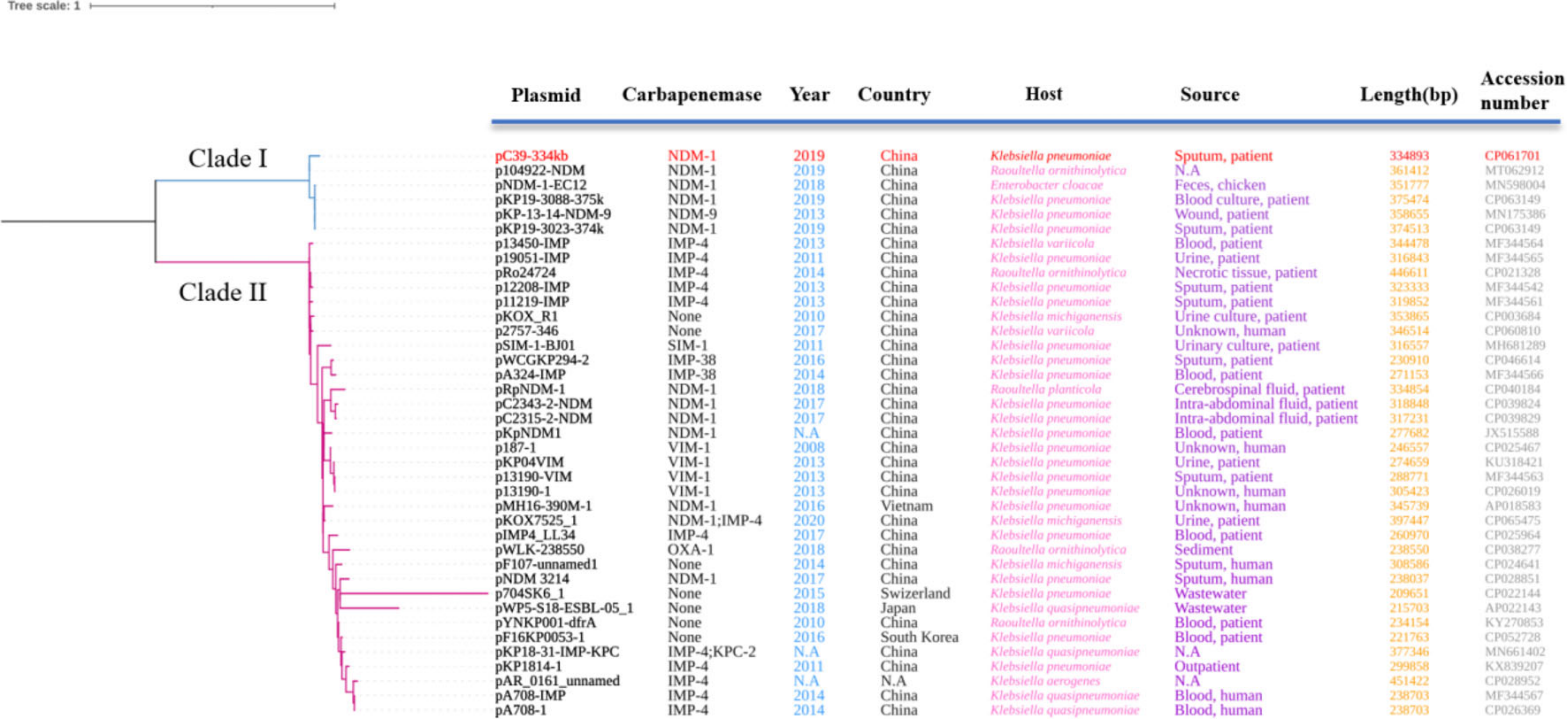
Phylogenetic tree of 39 plasmids constructed based on recombination-free SNPs. Clade I includes pC39-334kb with the five most homologous plasmids, among which pNDM-1-EC12 belongs to IncHI5 plasmids based on replicon genes. All plasmids contained in clade II are IncHI5 plasmids. The plasmid involved in this study is highlighted in red. N.A, not available.

To explore the phylogenetic relationship between IncHI5-like and IncHI5 plasmids, we downloaded complete sequences of 34 IncHI5 plasmids and 4 IncHI5-like plasmids. All plasmids could be assigned into two distinct clades. pC39-334kb with five homologous plasmids including IncHI5 plasmid pNDM-1-EC12 were clustered into clade I and separated from clade II which contained typical IncHI5 plasmids (**Figure 2**). These findings demonstrated that pNDM-1-EC12-like plasmids played as ancestral plasmids in the evolution of IncHI5-like plasmids. The mutation of replication genes probably resulted in the occurrence of IncHI5-like lineage, since pNDM-1-EC12 and all IncHI5-like plasmids are closely related genetically.

Plasmids were usually prone to mutations and rearrangements of certain sequences, which could also occur in the regions used for plasmid typing (Rozwandowicz et al., 2018), leading to the occurrence of novel untypeable plasmids or new plasmid lineages evolved from the well-studied plasmid types (Zhang et al., 2021). Furthermore, acquisition or loss of genes could be frequently detected in IncHI5 plasmids (Liang et al., 2018). These characteristics were the potential driving force of the evolution process of IncHI5 plasmids. Previously, a phylogenetic tree of 52 IncHI2 plasmids was constructed based on the SNPs of core plasmid genomes (Zhu et al., 2019). In consistent with our finding, all IncHI2 plasmids fell into two distinct clades, among which the plasmids from clade 1 differed greatly from those in clade 2, implying that the occurrence of two distinguished subtype plasmids in IncHI type plasmid is common. According to IncHI2 subtyping scheme (García-Fernández and Carattoli, 2010), it is necessary to propose the feasible plasmid double locus sequence typing scheme (pDLST) to detect the possible emergence and spread of IncHI5 subtype plasmids.

### MDR Region of pC39-334kb

**Figure 3:**
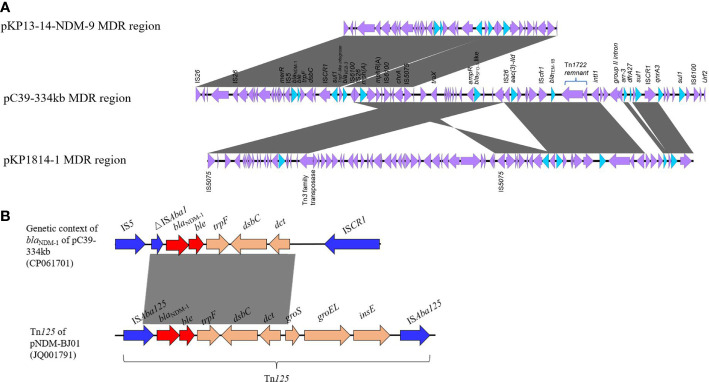
**(A)** Linear comparison of MDR regions among pC39-334kb, pKP13-14-NDM-9 (MN175386), and pKP1814-1 (KX839207). **(B)** Comparative analysis between genetic context of *bla*_NDM-1_ in pC39-334kb and Tn*125*. The sequence of Tn*125* is derived from pNDM-BJ01 (JQ001791) in *Acinetobacter lwoffii*.

A large ~68 kb MDR region was detected in pC39-334kb. Abundant AMR genes and mobile elements were concentrated in this region (**Figure 3A**). The *bla*_NDM-1_ gene was located in a structure of ΔIS*Aba125*-*bla*_NDM-1_-*ble*_MBL_-*trpF*-*dsbC*, which shared 100% nucleotide identity with the corresponding region of the transposon Tn*125* (**Figure 3B**), followed by IS*CR1*-*sul1*-*tnpF-like* integrase gene-*bla*_VEB-3_-IS*6100*. This segment was identical to that of pKP13-14-NDM-9 except for partial sequence loss of *tnpF-like* gene and *bla*_VEB-3_ gene, which was possibly caused by *tnpF-like* integrase (Huang et al., 2010). The following region, including IS*26*-*mph*(A)-*mrx*-*mphR-*IS*6100* unit, *chrA*, *bla*_SFO-1_-like (with three bases mutation compared to *bla*_SFO-1_) located adjacent to its regulator *ampR*, IS*26*-*aac(3)-IId-*IS*cfr1*-*bla*_TEM-1B_, and an incomplete Tn*1722* transposon, was most similar to the MDR region of pKP1814-1. However, the 25-kb region between IS*5075* and Tn*3* family transposase gene was absent in pC39-334kb and the IS*26*-*mph*(A)-*mrx*-*mphR-*IS*6100*-*chrA*-IS*5075* structure underwent the rearrangement of genetic position, in contrast to plasmid pKP1814-1. The last region was an archetypical complex class 1 integron with the structure of *intI1*-group II intron-*arr-3*-*dfrA27*-*qacE*Δ*1*-*sul1*-IS*CR1*-*qnrA3*-*qacE*Δ*1*-*sul1*. The formation of highly mosaic MDR region of pC39-334kb was derived from multiple insertion, deletion, and recombination events of AMR genes with various mobile elements.

### Transfer Regions tra1 and tra2 of pC39-334kb

To explore the underlying mechanism that accounted for the conjugation deficiency of pC39-334kb, the conjugative transfer region of untransferable plasmid pNDM-1-EC12 (Zhu et al., 2020) and transferable plasmid pIMP4_LL34 (Liu et al., 2018) were compared to that of pC39-334kb. It was worth noting that the IS elements and a toxin/antitoxin system RelB/StbD were inserted in the tra2 region in pC39-334kb. However, they did not truncate and inactivate *tra* genes. In the tra1 region, not only do the sequences of *traI* and *traG* genes of both untransferable plasmids have great difference with pIMP4_LL34, but the complete sequences of them also shared low similarity with pIMP4_LL34. Moreover, as mentioned in a previous study (Zhu et al., 2020), several *tra* genes, including *traM*, *traP*, *traQ*, *traR*, *traS*, *traT*, *traY*, and *traZ* were absent in pC39-334kb, which might be the underlying cause to impair the transferability of pC39-334kb (**Supplementary Figure S2**).

### Prevalence Characteristics of IncHI5-Like Plasmids

To get further insights into the prevalence characteristics of IncHI5-like plasmids, detailed information of the other four plasmids were investigated (**Table 2**). The results showed that four plasmids (pC39-334kb, pKP-13-14-NDM-9, pKP19-3023-374kb, and pKP19-3088-375kb) were found in *K. pneumoniae* from Chinese nosocomial setting. Surprisingly, the first occurrence of the IncHI5-like plasmid could date back to 2013. For nearly a decade, it was not growing in rapid trend, which was largely due to the fact that IncHI5-like plasmids were not capable of transfer by conjugation. Furthermore, IncHI5-like plasmids were important vectors associated with MDR phenotype; in particular, the *bla*_NDM_ gene was detected in all IncHI5-like plasmids. Therefore, even though the plasmid was unable to transfer, recalcitrant selection pressure could potentially contribute to the maintenance and spread of IncHI5-like plasmids (Baker-Austin et al., 2006). Five IncHI5-like plasmids ranged from ~334 to ~375 kb in size and carried a typical MDR region comprising various accessory and AMR genes. However, the MDR regions of five plasmids were distinct. Some potential insertion hot spots were detected in backbone regions of IncHI5-like plasmids. Examples included the *umuC* gene, the locus between two genes encoding IS*3* family transposase and Na^+^/H^+^ antiporter (**Supplementary Figures S3** and **S4**). These insertion hot spots could be regarded as candidate loci for foreign genes, thus facilitating the evolution of plasmid. There were a total of 530 genes consisting of core genes and accessory genes. Among them, 262 genes were found to be shared by all plasmids as core genes encoding replication, maintenance, and hypothetical genes of unknown functions, whereas the remaining 268 genes were accessory genes, which were mainly composed of resistance genes, insertion sequences, and toxin/antitoxin system (**Supplementary Figure S5**).

**Table 2:** Basic information of five IncHI5-like plasmids investigated in this study.

	AMR genes	Year	Country	Source	Host species	Lengths	Accession number
pC39-334kb	*qnrA7, aac(3)-IId, bla*_SFO-1_-like, *bla*_VEB-3_, *bla*_TEM-1B_, *bla*_NDM-1,_ *arr-3, mph*(A)*, sul1, dfrA27, ble*	2019	China	Sputum; patient	*Klebsiella pneumoniae*	334,893 bp	CP061701
pKP-13-14-NDM-9	*bla*_CTX-M-14_, *tet*(D), *sul2*, *strA*, *strB*, *bla*_TEM-1B_, *aac(3)-IId, dfrA13, aadA2, sul1,mph*(A), *bla*_NDM-1_	2013	China	Wound; patient	*Klebsiella pneumoniae*	358,655 bp	MN175386
pKP19-3023-374k	*tmexCD1- toprJ1, sul2, strA, strB, bla*_TEM-1B,_ *aac(3)-IId, mph*(A), *sul1*, *qnrB6*, *aadA16, dfrA27, arr-3, bla*_NDM-1_	2019	China	Sputum; patient	*Klebsiella pneumoniae*	374,513 bp	CP063748
pKP19-3088-375k	*tmexCD1- toprJ1, sul2, strA, strB, bla*_TEM-1B,_ *aac(3)-IId, mph*(A), *sul1, qnrB6, aadA16, dfrA27, arr-3, bla*_NDM-1_	2019	China	Blood culture; patient	*Klebsiella pneumoniae*	375,474 bp	CP063149
p104922-NDM	*bla*_CTX-M-14_, *tet*(D), *catA2, sul2, strA, strB*, *bla*_TEM-1B_, *dfrA12, aadA2, sul1, bla*_NDM-1_, *msr*(E), *mph*(E)	2019	China	Unknown	*Raoultella ornithinolytica*	361,412 bp	MT062912

## Conclusions

To conclude, this study identified a *bla*_NDM-1_-bearing IncHI5-like plasmid in *K. pneumoniae* from an infant patient. As emerging plasmids in China, IncHI5-like plasmids have separated from typical IncHI5 plasmids and have the potential to evolve into an important vector for the spread of AMR genes. Despite the fact that this type of plasmid was nonconjugative, co-selection could potentially result in consistent maintenance and spread of IncHI5-like plasmid due to the existence of rich AMR genes. The prevalence of *bla*_NDM_-carrying IncHI5-like plasmids among various pathogens in clinical and other settings warrants further investigations, and the evolution pathway of the plasmid should be closely tracked in the future.

## Data Availability Statement

The complete sequences of C39 were deposited in GenBank with the accession numbers CP061700 (chromosome), CP061701 (pC39-334kb) and CP061702 (pC39-125kb), respectively.

## Author Contributions

Supervision and conceptualization: ZW and RL. Original draft preparation and visualization: ZL. Methodology, investigations, and resources: RC and PX. All authors contributed to the article and approved the submitted version.

## Funding

This work was supported by the Natural Science Foundation of Jiangsu Province (BK20180900), Joint Co-construction Project of Henan Medical Science and Technology Research (LHGJ20200073), and the Priority Academic Program Development of Jiangsu Higher Education Institutions (PAPD).

## Conflict of Interest

The authors declare that the research was conducted in the absence of any commercial or financial relationships that could be construed as a potential conflict of interest.

## Publisher’s Note

All claims expressed in this article are solely those of the authors and do not necessarily represent those of their affiliated organizations, or those of the publisher, the editors and the reviewers. Any product that may be evaluated in this article, or claim that may be made by its manufacturer, is not guaranteed or endorsed by the publisher.
