# Supplementary material for: Characterization of a bla NDM-1-Bearing IncHI5-Like Plasmid From Klebsiella pneumoniae of Infant Origin
Source: Front Cell Infect Microbiol. 2021 Oct 1;11:738053. doi: 10.3389/fcimb.2021.738053 (PMC8517479; doi:10.3389/fcimb.2021.738053)
Supplement: Supplementary file 1 [file DataSheet_1.docx]

**Supplementary Data**

**Table S1** Basic information and genomic characterization of C39 revealed by WGS data.

|  | Species | MLST/serotype | Isolate date | Patient basic information | Diagnosis | Patient with symptoms | Medication history | Constituent | Size(bp) | Number of resistance genes | Accession numbers |
| --- | --- | --- | --- | --- | --- | --- | --- | --- | --- | --- | --- |
| C39 | *K. pneumoniae* | ST37/KL15 | 08/10/2019 | A male infant patient | Congenital heart disease | Tussicula Asthma | Imipenem | Chromosome | 5,290,020bp | *oqxA, oqxB, bla*_SHV,_ *fosA,* | CP061700 |
|  |  |  |  |  |  |  |  | pC39-334kb (IncHI5-like) | 334,893bp | *qnrA7, aac(3)-IId, bla*_SFO-1_, *bla*_VEB-3_, *bla*_TEM-1B_, *bla*_NDM-1_, *arr-3, mph(A),sul1,dfrA27,ble* | CP061701 |
|  |  |  |  |  |  |  |  | pC39-125kb (IncFIB) | 125,663bp | - | CP061702 |


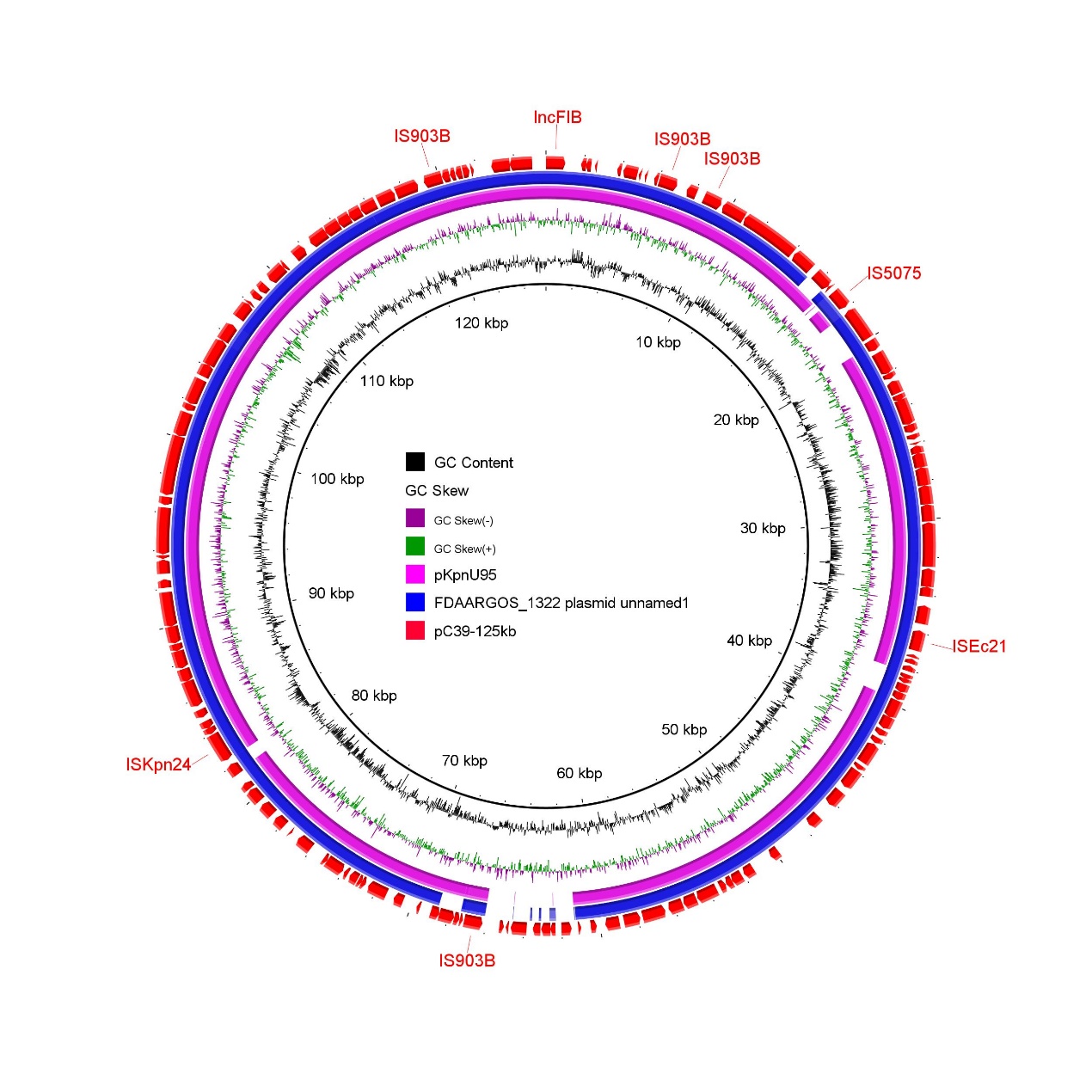
 **Figure S1.** Circular comparison of pC39-125kb and other two similar plasmids in NCBI database.


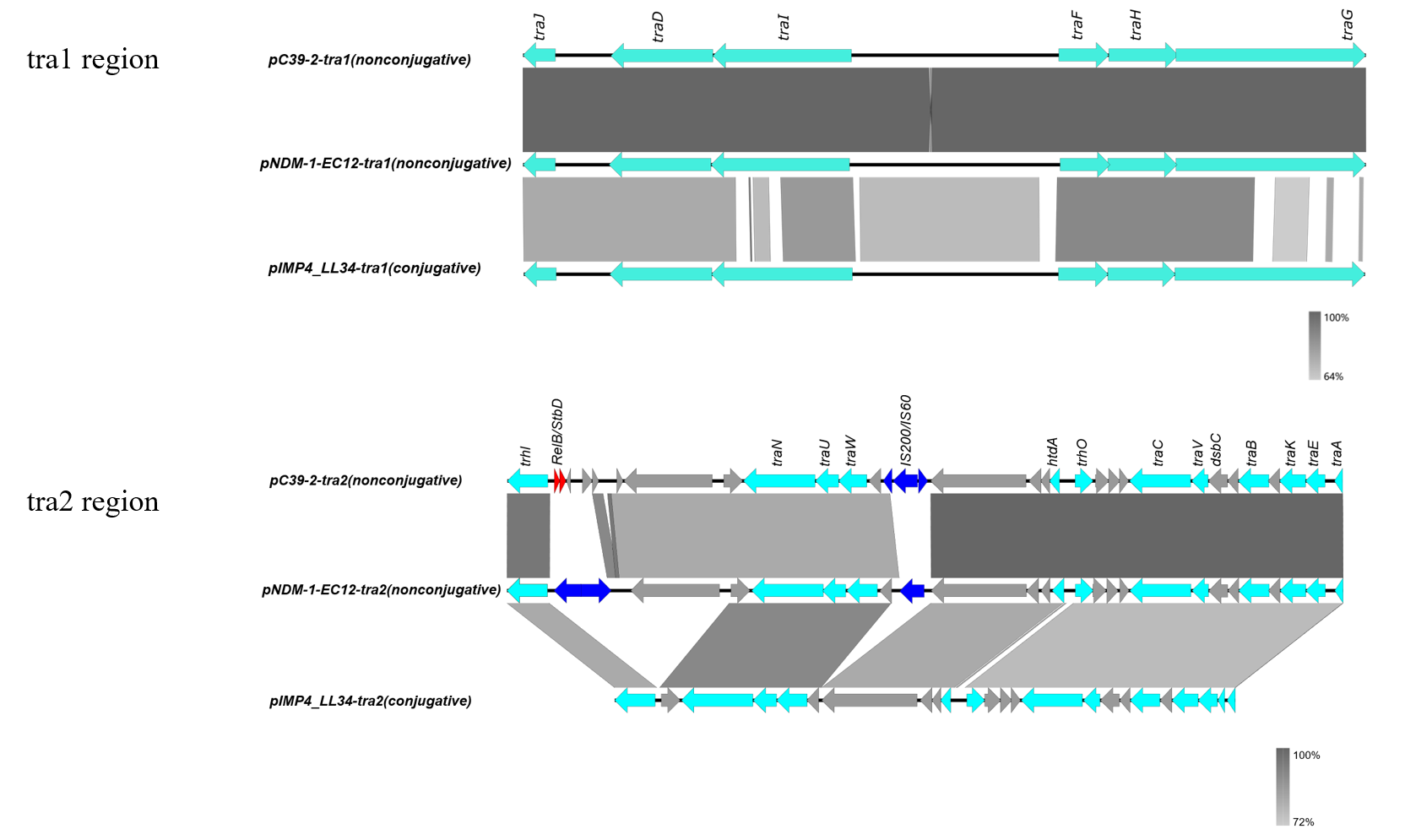


**Figure S2.** Comparative analysis of tra1 and tra2 regions of pC39-334kb with pNDM-1-EC12(nonconjugative, MN598004) and pIMP4_LL34 (conjugative, CP025964). The *tra* genes, mobile elements, toxin/antitoxin system and other genes were represented by light green, blue, red, and grey arrows, respectively.


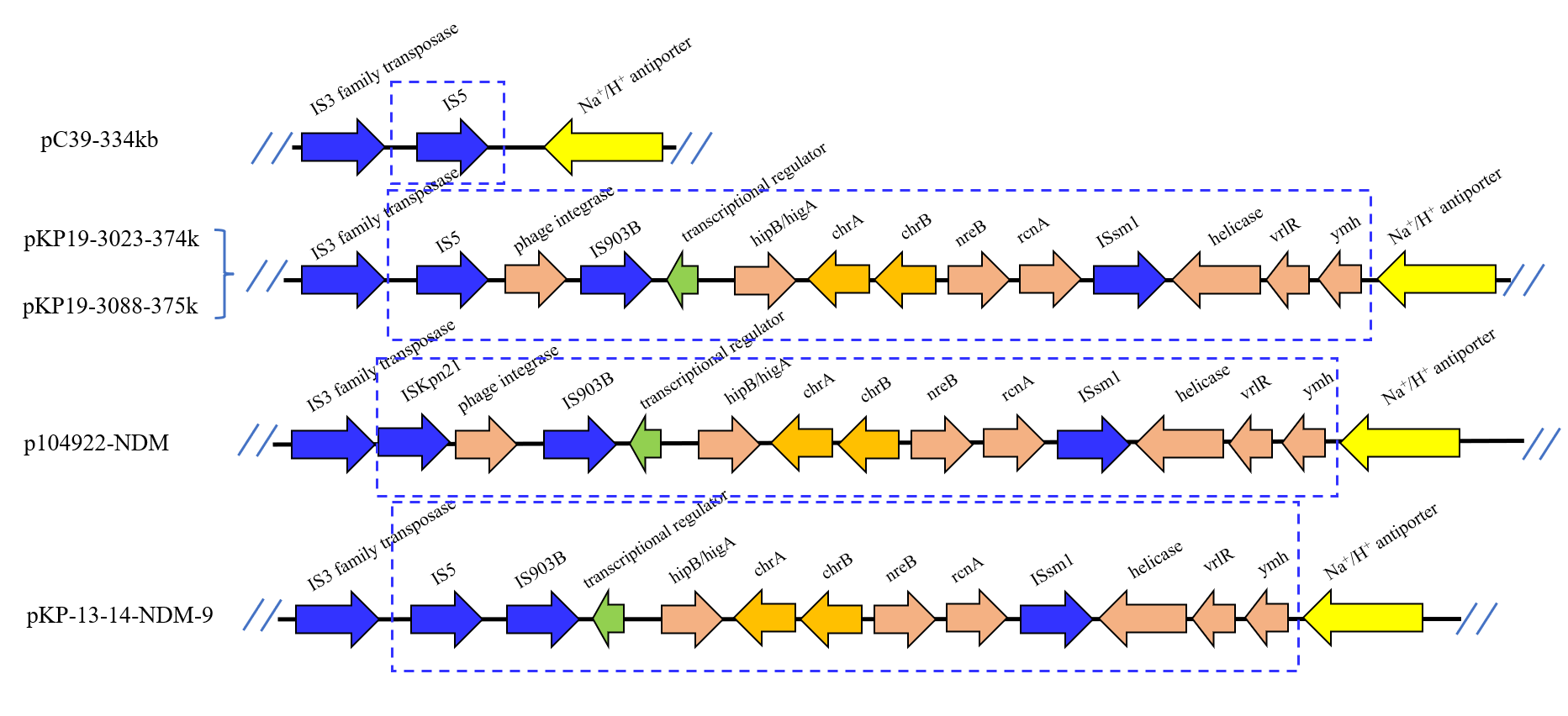
 **Figure S3.** Arrangement of functional genes between IS*3* family and Na+/H+ antiporter genes in different IncHI5-like plasmids.


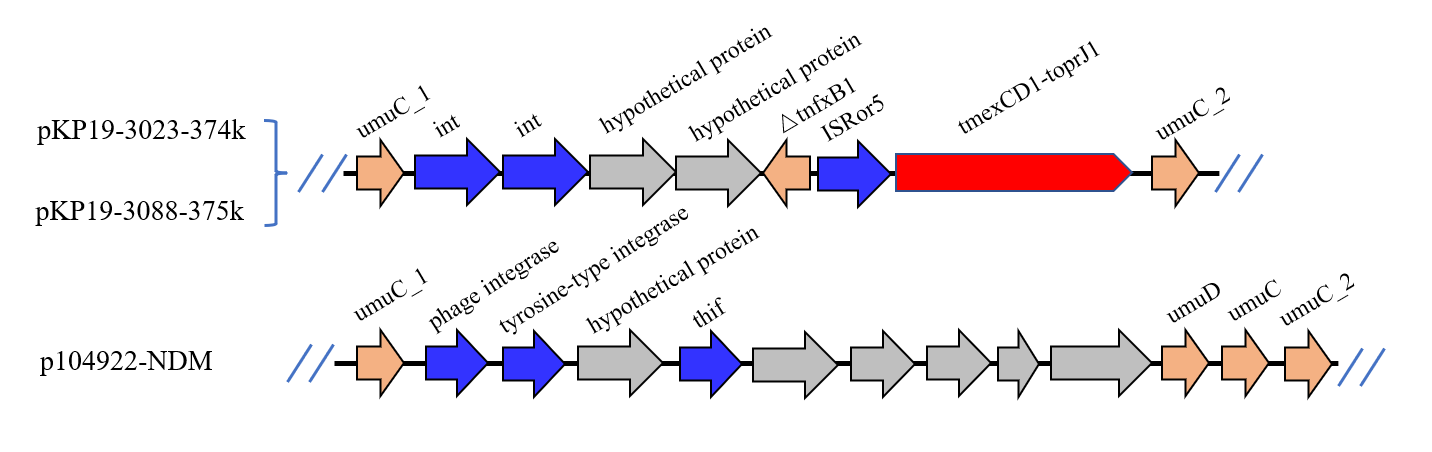
 **Figure S4.** Complete structures between insertion hot spot *umuC* gene in different IncHI5-like plasmids.


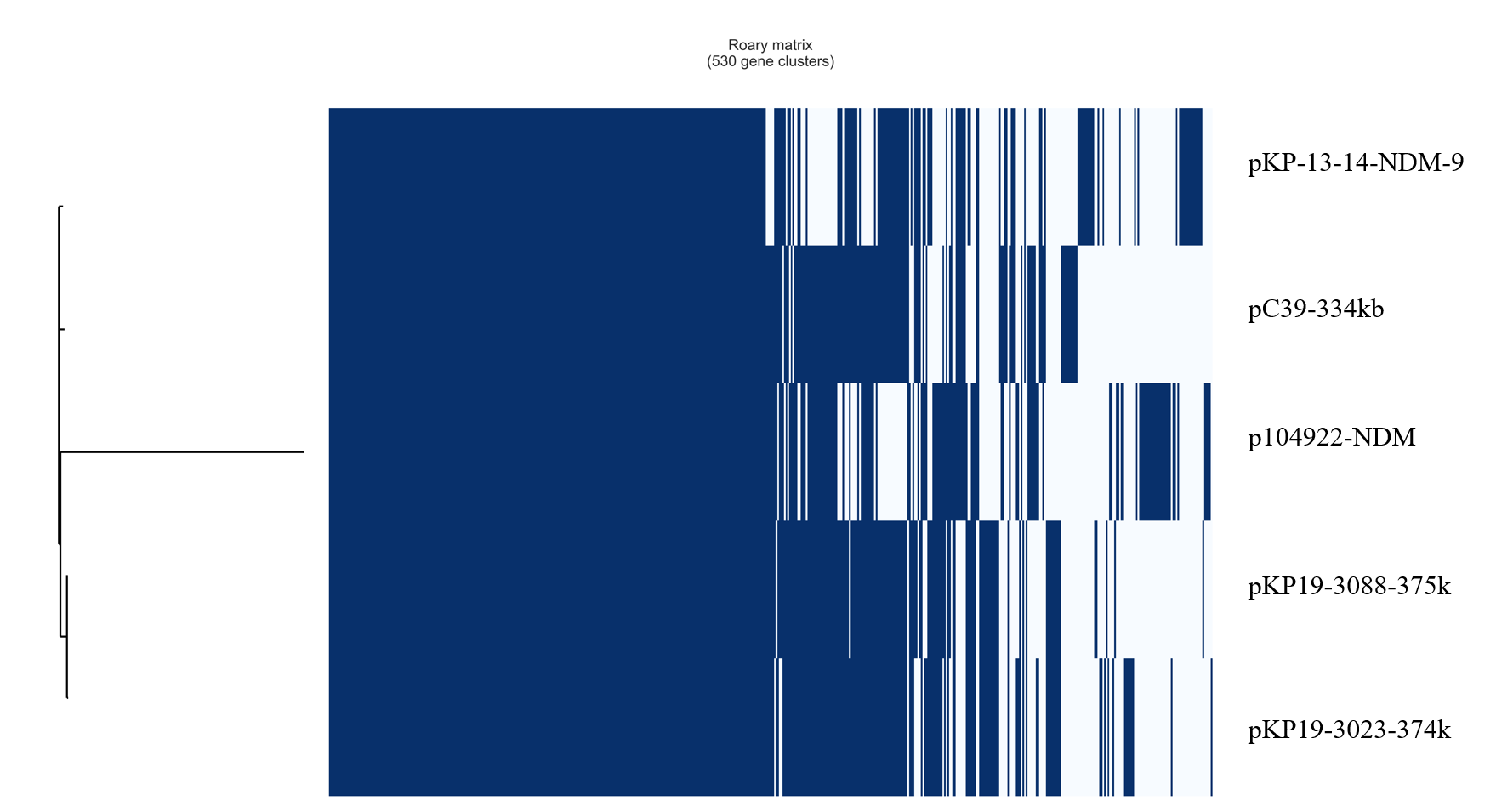


**Figure S5.** Pangenome analysis of five IncHI5-like plasmids revealed by Roary. The blue bar indicated the pangenome of all IncHI5-like plasmids, including the 530 annotated genes in total.
